# Outcome and Prognostic Factors of Dogs Treated for Infiltrative Lipoma Undergoing Radiation Therapy: A Retrospective Multi‐Institutional Study of 29 Cases

**DOI:** 10.1111/vco.13065

**Published:** 2025-05-14

**Authors:** Arata Matsuyama, Valerie J. Poirier, Michelle M. Turek, Valeria S. Meier, Jessica Lawrence

**Affiliations:** ^1^ Department of Small Animal Clinical Sciences Western College of Veterinary Medicine, University of Saskatchewan Saskatoon Saskatchewan Canada; ^2^ Department of Biomedical Sciences Ontario Veterinary College, University of Guelph Guelph Ontario Canada; ^3^ Department of Clinical Studies Ontario Veterinary College, University of Guelph Guelph Ontario Canada; ^4^ Department of Surgical Sciences School of Veterinary Medicine, University of Wisconsin‐Madison Madison Wisconsin USA; ^5^ Clinic of Radiation Oncology and Medical Oncology, Small Animal Department Vetsuisse Faculty, University of Zurich Zurich Switzerland; ^6^ Department of Veterinary Clinical Sciences College of Veterinary Medicine, University of Minnesota St Paul Minnesota USA; ^7^ Department of Surgical and Radiological Sciences School of Veterinary Medicine, University of California Davis California USA

**Keywords:** canine neoplasms, multicenter study, prognosis, radiotherapy dosage, veterinary oncology

## Abstract

Canine infiltrative lipomas are localised and invasive tumours that are commonly treated with surgery and/or radiation therapy (RT). There is limited efficacy data for treatment of infiltrative lipomas, and optimal fractionation strategies remain unclear. We retrospectively assessed the outcomes and prognostic factors in a cohort of dogs that underwent intensity modulated or three‐dimensional conformal RT for infiltrative lipoma. Twenty‐nine dogs were included from four academic institutions. The median total radiation dose prescribed and delivered was 51 Gy (range = 20–57 Gy). Dose per fraction ranged from 2.4 to 4.2 Gy, with a median of 3 Gy. Median progression‐free survival (PFS) was 1483 days; the median overall survival (OS) was 1483 days. Disease progression was documented in four dogs (14%), all of which received less than 51 Gy (range = 20–50 Gy). Grade V adverse events (AEs) or secondary malignancies were recorded in six dogs (21%; two bones, one skin, one lungs, one urethra and one small intestine AE); assigned attribution was definite (*n* = 1), probable (*n* = 2), possible (*n* = 2) and unlikely (*n* = 1). Gross tumour volume (GTV) was prognostic for PFS (*p* < 0.01) while both GTV (*p* < 0.01) and total radiation dose (*p* < 0.01) were prognostic for OS (*p* < 0.01). The number of surgeries and tumour location were not associated with PFS or OS. These findings support the use of RT for long‐term local control for unresectable or incompletely excised canine infiltrative lipomas. A higher total radiation dose may result in better long‐term local disease control.

## 1 Introduction

Infiltrative lipoma is a rare but locally invasive form of lipoma in dogs [1]. Complete surgical resection is the standard of care treatment for the highest likelihood of long‐term local tumour control. Complete surgical margins can be challenging, particularly for larger or diffusely located disease or for tumours on the extremities, and local recurrence is common following incomplete excision and can lead to mobility issues or life‐threatening clinical signs [1, 2, 3]. In one study of five dogs with recurrent infiltrative lipoma, the median time to recurrence was 239 days [3].

Based on limited data, radiation therapy (RT) is an effective alternative treatment for dogs with incompletely resected infiltrative lipomas or for dogs that are not candidates for surgical resection. In an early study of 13 dogs prescribed a total dose ranging from 45.6 to 63 Gy with ^60^Cobalt, with or without hyperthermia, the median overall survival (OS) was approximately 1200 days [4]. Notably, among the nine dogs with measurable gross disease, all but one had an objective response (*n* = 4) or stable disease (*n* = 4). Most recently, Feng et al. reported excellent, long‐term survival in 24 dogs prescribed a total dose of 45–51 Gy with a median fraction size of 3 Gy [5]. The median OS exceeded 4 years, and none of the dogs were reported to have died due to infiltrative lipoma. Additionally, Hauser et al. reported a median OS of 1694 days in 21 dogs prescribed 45–54 Gy with a median fraction size of 3 Gy [6]. Five of these 21 dogs (24%) developed local recurrence or disease progression. In that study, the number of prior surgeries was associated with tumour control, although OS was not impacted. While RT appears to be an effective therapy for canine infiltrative lipomas, the existing literature is limited. Additional data are needed to determine ideal radiotherapy case selection, clinical prognostic factors and fractionation techniques. The purpose of this study was to analyse clinical and radiotherapy prognostic variables for dogs with infiltrative lipomas treated with RT.

## 2 Materials and Methods

### 2.1 Study Design

This is a retrospective multi‐institutional study. Cases were recruited via the American College of Veterinary Radiology listserv (recruitment announcement made in May 2022). Inclusion criteria included dogs with a histological diagnosis of infiltrative lipoma or a cytologic diagnosis of lipoma and evidence of a fat opacity mass with invasion into adjacent tissues such as skeletal or body wall muscles on computed tomography (CT) [7], and treatment with intensity modulated RT (IMRT) or three‐dimensional conformal RT (3DCRT) between January 2013 and December 2019. Dogs with primary, recurrent macroscopic or microscopic tumours were included provided there was a minimum follow‐up time of 2 years, unless death or disease progression was documented.

### 2.2 Data Collection

Medical records for cases that met the inclusion criteria were reviewed for the following factors at the time of RT: sex, age, breed, body weight, comorbidities, clinical signs, tumour location, number of prior surgeries, types of diagnostic imaging performed, diagnostic confirmation methods (cytology or histopathology), disease setting (macroscopic or microscopic) and maximum tumour diameter.

Radiation treatment details, including protocol, contouring expansion approaches, planned doses to gross tumour volume (GTV), clinical target volume (CTV), planning target volume (PTV), organs at risk (OAR) and use of bolus were retrieved. Details regarding the planning and treatment delivery systems were recorded. Adverse events (AEs) were categorised as acute or late based on the timing relative to RT completion (before or after 3 months) and graded retrospectively based on Veterinary Radiation Therapy Oncology Group (VRTOG) toxicity criteria v2.0 [8]. The current VRTOG guidelines do not include radiation‐induced malignancies as an AE, in line with current CTCAE, RTOG and LENT‐SOMA scales for humans (REFS) [8, 9, 10, 11]. For the purposes of this study and following consensus among the participating radiation oncologists in this study, malignancies that developed within the RT field that led to euthanasia were considered Grade V AEs. Attribution was applied based on group consensus. Details regarding follow‐up, re‐imaging, adjuvant therapy, objective progression and cause of death were recorded when available. The retrospective, multi‐institutional study design and lack of standardised follow‐up precluded objective tumour response assessment. When available survival information was insufficient, the primary veterinarians and/or pet owners were contacted for follow‐up information.

### 2.3 Statistical Analysis

Descriptive and analytical statistics were performed. Descriptive statistics consisted of median values for continuous data and frequencies for categorical data. The Kaplan–Meier method was used for survival analysis. OS was defined as the duration from the first RT date to death/euthanasia of any cause; dogs that were alive at the time of data collection or lost to follow‐up were censored. Progression‐free survival (PFS) was defined as the duration from the first RT date until tumour progression or death/euthanasia of any cause; dogs that were alive at the time of data collection without disease progression were censored. Log‐rank tests were used for survival comparison based on different disease status (microscopic vs. macroscopic disease), presence of clinical signs at the time of RT other than the presence of a mass (yes vs. no), presence of comorbidities (yes vs. no), tumour location (limb; head; limb to trunk; thoracic wall), number of previous surgeries (0 vs. 1 vs. 2 vs. 3; 0 vs. 1–3), as well as total radiation dose with the median value as a cut‐off. A Cox proportional‐hazards model was also used to assess the effect of the following factors on OS and PFS: disease status (microscopic or macroscopic disease), presence of clinical signs at the time of RT, presence of comorbidities, tumour location, number of previous surgeries, GTV in dogs with gross disease and total radiation dose. Variables were evaluated with univariate analysis; factors with a *p* < 0.1 were included in multivariate analysis. A *p* value < 0.05 was considered statistically significant in the multivariate analysis. Statistical analyses were performed using SPSS version 23.0 (SPSS Inc., Chicago, IL).

### 2.4 Cell Line Authentication Statement

No cell lines were used in the current study.

## 3 Results

### 3.1 Patient Characteristics

Twenty‐nine cases from Ontario Veterinary College (*n* = 16), University of Wisconsin (*n* = 7), University of Zurich (*n* = 4) and University of Minnesota (*n* = 2) met the inclusion criteria. The baseline patient characteristics are summarised in Table 1. Clinical signs varied, but the majority (*n* = 22, 76%) presented with a palpable mass. Additional clinical signs at presentation included lameness (*n* = 3), limb swelling (*n =* 1), abnormal limb extension (*n* = 1), post‐operative seroma (*n* = 1) and tenesmus (*n* = 1). The median interval between the onset of initial signs and RT in the 26 cases from which data were available was 270 days (range = 20–1095 days). Of these, seven dogs underwent RT as a primary therapy with a median interval of 56 days (range = 20–730 days). Nine cases (31%) presented with comorbidities, including either or combinations of atopic dermatitis, brachycephalic airway syndrome, chronic cholangiohepatitis with biliary hyperplasia and hepatic fibrosis, surgically excised hemangiopericytoma, unilateral renal agenesis and splenic low‐grade lymphoma.

**TABLE 1:** Baseline characteristics of 29 dogs.

Case	RT	Sex	Age (year old)	Breed	Body weight (kg)	Tumour location	Pre‐RT surgeries	Disease setting at RT	Size (cm)
1	IMRT	FS	5	Bernese Mountain Dog	48	Thoracic wall	2	Macro	9.8
2	IMRT	MC	9	Jack Russell Terrier	10.2	Limb	0	Macro	6.7
3	IMRT	MC	6	Miniature Schnauzer	12.8	Thoracic wall	2	Micro	N/A
4	IMRT	MC	4	Shetland Sheepdog	9.6	Limb	1	Macro	5
5	IMRT	MC	8	Catahoula Leopard Dog	34.7	Limb	0	Macro	16.7
6	IMRT	FS	8	English Bull Terrier	22.4	Head	1	Macro	8
7	IMRT	FS	4	Labrador Retriever	36.6	Thoracic wall	2	Micro	N/A
8	IMRT	FS	8	Catahoula Leopard Dog	26.8	Limb	2	Micro	N/A
9	IMRT	FS	4	Mixed breed	30.2	Thoracic wall	1	Micro	N/A
10	IMRT	MC	4	Maltese	7.7	Limb	1	Macro	3.8
11	IMRT	FS	6	Golden Retriever	37.8	Thoracic wall	1	Macro	10.3
12	IMRT	MC	4	Mixed breed	31.4	Thoracic wall	2	Micro	N/A
13	IMRT	MC	3	Dalmatian	21.4	Thoracic wall	0	Macro	10.7
14	IMRT	FS	9	Vizsla	26.2	Limb	0	Macro	9
15	IMRT	FS	7	Labrador Retriever	37.5	Limb	3	Macro	20.9
16	IMRT	MC	4	French Bulldog	11.8	Head	0	Macro	4.9
17	3DCRT	FS	7	Cocker Spaniel	11.7	Limb to trunk	1	Macro	8.1
18	IMRT	MC	6	Boxer	37.8	Limb to trunk	0	Macro	20.2
19	3DCRT	F	8	Collie	21.5	Head	1	Macro	Diffuse
20	3DCRT	M	5	Mixed breed	47.8	Limb	1	Macro	8.2
21	IMRT	MC	6	Border Terrier	8.9	Limb	1	Macro	4.6
22	IMRT	FS	5	Cavalie King Charles Spaniel	7.4	Limb	0	Macro	9.9
23	IMRT	MC	7	Pomeranian	6.5	Limb	1	Macro	6
24	IMRT	MC	3	Mixed breed	8.8	Limb	1	Macro	9
25	IMRT	FS	10	Fox Terrier	9.4	Limb to trunk	2	Micro	N/A
26	IMRT	MC	10	Pug	5.9	Limb to trunk	1	Macro	7.7
27	IMRT	FS	7	Siberian Husky	27.5	Head	3	Macro	8
28	IMRT	MC	6	Labradoodle	39.4	Limb	1	Macro	3
29	IMRT	FS	9	Mixed breed	23.2	Limb	1	Macro	4.8

Abbreviations: 3DCRT, three‐dimensional conformal RT; F, female; FS, female spayed; IMRT, intensity modulated RT; M, male; MC, male castrated; Macro, macroscopic disease; Micro, microscopic disease; N/A, not applicable; RT, radiation therapy.

Tumours were located on the limb (*n* = 14), thoracic wall (*n* = 7), head (*n* = 4) or limb to trunk (*n* = 4). Seven dogs (24%) underwent RT as a primary treatment, while 22 dogs (76%) underwent surgical resection of infiltrative lipoma prior to RT either once (*n* = 14), twice (*n* = 6) or three times (*n* = 2). Diagnosis was confirmed by histopathology (*n* = 25; 86%) or cytology (*n* = 4; 14%) in conjunction with CT (*n* = 29; 100%). Macroscopic tumours were irradiated in 23 dogs (79%), and microscopic tumours were irradiated in 6 dogs (21%). The median maximum diameter in 22 dogs with macroscopic disease was 8 cm (range = 3–21 cm); one tumour was diffuse and could not be accurately measured. Staging included thoracic imaging in 27 dogs (93%) with CT (*n* = 16) or radiographs (*n* = 11), and abdominal imaging in 11 dogs (38%) with CT (*n* = 3) or ultrasound (*n* = 8).

### 3.2 RT

A total of 26 dogs were treated with IMRT, while 3 received 3DCRT. Treatments were delivered using a Clinac iX linear accelerator (Varian Medical Systems, Palo Alto, CA) or TomoTherapy HiArt Treatment System (Accuray Inc., Sunnyvale, CA). Planning systems included Eclipse v11, v13 or v15 (Varian Medical Systems), TomoTherapy HiArt v3‐5 (Accuray Inc.) and Pinnacle v.9.10 (Philips NV, Amsterdam, the Netherlands).

Radiation treatment was administered daily from Monday to Friday in all dogs with various fractions (Fr). Fractionation schemes included 3 Gy × 19 Fr (*n* = 8), 3 Gy × 17 Fr (*n* = 4), 2.5 Gy × 20 Fr (*n* = 3), 2.67 Gy × 18 Fr (*n* = 3), 2.7 Gy × 18 Fr (*n* = 3), 4 Gy × 5 Fr (*n* = 2), 3 Gy × 19 Fr (*n* = 2), 2.4 Gy × 20 Fr (*n* = 1), 2.6 Gy × 20 Fr (*n* = 1), 2.8 Gy × 19 Fr (*n* = 1) and 4.2 Gy × 10 Fr (*n* = 1). One dog was prescribed 2.8 Gy × 19 Fr but only a total dose of 36.4 Gy was given due to AE. The median total radiation dose received was 51 Gy (range = 20–57 Gy), with three dogs receiving < 40 Gy (4 Gy × 5 Fr and 2.8 Gy × 13 Fr). For the two dogs that received the 4 Gy × 5 Fr protocol, both had gross disease located on the limb extending to the trunk, with GTV of 1596.1 and 50.5 cm^3^, respectively. One dog presented without any clinical signs other than the presence of a mass, while the other dog was a Pug with brachycephalic airway syndrome that presented with tumour‐associated tenesmus.

Tumour and target volumes for RT are summarised in Table 2. Contours for GTV were delineated based on visible (gross) abnormal fat identified on CT (*n* = 23) and surgical incision (*n* = 2) with hemoclips (*n* = 4). CTV was contoured by extending GTV to anatomical boundaries/fascia with a median axial expansion of 3 cm (range = 1–4.5 cm) in 28 dogs, with 1 dog having CTV equivalent to GTV. PTV was contoured with a median isotropic expansion of 5 mm (range = 0.5–7 mm) from CTV. Bolus was utilised in 23 dogs to improve dose homogeneity within the target volumes. OARs and dose statistics are summarised in Table S1.

**TABLE 2:** Tumour and target volumes calculated for radiation therapy.

	*n*	Median	Min	Max
GTV (cm^3^)	29^a^	154.21	1.8	2534
	23^b^	145	5.7	2534
GTV D50 (Gy)	29^a^	54.8	20.2	59.2
	23^b^	54	20.2	59.2
GTV D95 (Gy)	29^a^	54.2	19.7	57.4
	23^b^	52.1	19.7	57.4
GTV D98 (Gy)	29^a^	53.2	19.9	56.9
	23^b^	51.8	19.85	56.9
GTV D2 (Gy)	29^a^	57.4	20.4	104.6
	23^b^	54.9	20.4	61
PTV (cm^3^)	29	621.3	106.5	3550.1
PTV D50 (Gy)	29	52.4	20.2	58.9
PTV D95 (Gy)	29	48.7	19.7	55.1
PTV D98 (Gy)	29	47.6	15.4	54.4
PTV D2 (Gy)	29	56.3	20.5	62.1
CTV (cm^3^)	28^c^	356.85	78.9	2767.94

Abbreviations: CTV, clinical target volume; GTV, gross tumour volume; PTV, planning target volume.

^a^ GTV for microscopic disease was contoured on surgical incision with hemoclips.

^b^ Includes only dogs with gross disease at time of RT.

^c^ Combined CTV/GTV was used in one case.

Modifications to the intended RT protocol were made in six cases. In one dog, the bolus was removed at the 15th out of 20 Fr of 2.7 Gy, and a new treatment plan was created for the remaining 5 Fr to prevent worsening of Grade II radiation dermatitis. Two dogs receiving 18 or 20 Fr of 2.4–2.67 Gy, 2 Fr were given in 1 day 6 h apart due to a holiday or machine failure. One of these two dogs also required a plan revision following 15 out of 18 Fr due to a reduction in post‐operative swelling at the RT site. One dog developed a mast cell tumour at the edge of the RT field, and the treatment plan was modified following 15 out of 18 Fr to palliate the mast cell tumour. One dog was prescribed 2.8 Gy × 19 Fr but discontinued treatment after the 13th Fr due to inappetence and regurgitation secondary to Grade IV esophagitis. This dog was being treated for an 8 cm recurrent infiltrative lipoma in the submandibular region. Endoscopic findings at the onset of symptoms were consistent with severe radiation‐induced epiglottitis and esophagitis, while thoracic radiographs showed aspiration pneumonia. Consequently, the dog was successfully treated with supportive care, including placement of a per endoscopic gastrostomy tube, and RT was not resumed. In one dog, plan revision was made after 2 out of 18 Fr, but the rationale was not documented.

### 3.3 AEs

Acute and late AEs were reported in 28 and 26 dogs, respectively, and are summarised in Table 3. OAR dose statistics are shown in Table S1. Eight dogs (27%) experienced Grade III or higher acute AEs, and eight dogs (28%) developed Grade III or higher late AEs, including six dogs (21%) that were euthanised because of suspected Grade V AEs or malignancies within the RT field, as summarised in Table 4.

**TABLE 3:** Recorded radiation‐induced adverse events (Veterinary Radiation Therapy Oncology Group toxicity criteria v2.0 [8]).

Acute			Late		
None		*n* = 2	None		*n* = 10
Grade I	Skin	*n* = 8	Grade I	Skin	*n* = 8
Grade II	Skin	*n* = 10		Pain	*n* = 1
	Pain	*n* = 3	Grade II	Muscle/bone	*n* = 2
	Mucositis	*n* = 2	Grade III	Nerve	*n* = 2
	Cystitis	*n* = 1	Grade V	Skin/lung/bone/urethra/small intestine	*n* = 6
Grade III	Skin	*n* = 4			
	Pain	*n* = 2			
	Eye	*n* = 1			
Grade IV	Skin	*n* = 1			
	Pharynx and oesophagus	*n* = 1			

*Note*: Acute and late adverse events were reported in 28 and 26 dogs, respectively. Late Grade V adverse events included secondary malignancies as well.

**TABLE 4:** Summary of six cases with Grade V adverse events or secondary malignancies.

Dog	Outcome	Treated anatomic site	Attribution	Dose distribution
1	Non‐traumatic bone fracture	Semitendinosus and biceps femoris muscles	Probable	Femur: D50% 55.6 Gy; D98% 51.1 Gy; D2% 57.5 Gy
2	Progressive diffuse oedema and serous discharge within RT field	Ventral abdomen to medial thigh	Probable	Not available
3	Osteolysis of humerus with pulmonary nodules^a^	Axilla	Definite	Humerus: D50% 51.9 Gy; D98% 1.6 Gy; D2% 59.8 Gy
4	Post‐operative intestinal perforation following resection of recurrent infiltrative lipoma	Perianal area with pelvic and intra‐abdominal extension	Unlikely	Small intestine: D50% 12 Gy; D2% 42 Gy
5	Urethral urothelial carcinoma^a^	Medial thigh	Possible	Urethra: D50% 26.2 Gy; D2% 52.3 Gy.
6	Pulmonary carcinoma^a^	Shoulder	Possible	Lungs: D50% 15 Gy; D2% 52.7 Gy

^a^ Suspected secondary malignancy.

The majority (*n* = 26; 93%) of dogs evaluated following completion of RT developed at least one acute AE. The highest grade among the 26 dogs was I (*n* = 6), II (*n* = 12), III (*n* = 6) and IV (*n* = 2). Late AEs were less commonly seen but were documented as Grades I (*n* = 7), II (*n* = 2), III (*n* = 2) or V (*n* = 6). No late AEs were documented in 10 dogs. In addition, one dog developed incidentally detected, non‐painful tooth decay within the RT field that was suspected as a late AE. No grade was given due to the lack of a dental AE category in the existing grading scheme [8]. Protocols used in the eight dogs with Grade III or IV acute AEs include 3 Gy × 19 Fr (*n* = 3), 2.6 Gy × 20 Fr (*n* = 1), 3 Gy × 17 Fr (*n* = 1) and 2.67 Gy × 18 Fr (*n* = 1) for Grade III and 2.8 Gy × 19 Fr (*n* = 1; only 13 Fr administered) and 3 Gy × 19 Fr (*n* = 1) for Grade IV. The two dogs with Grade III late AEs received 3 Gy × 17 Fr and 2.4 Gy × 20 Fr protocols.

Six dogs (21%) died due to suspected Grade V late AEs or new malignancies within the RT field (Table 4). The first dog underwent 2.8 Gy × 19 Fr (IMRT) for a gross infiltrative lipoma measuring 16.7 cm in its longest diameter within the left semitendinosus and biceps femoris muscles. The recorded best tumour response was stable disease based on physical examination, but the dog presented to a local emergency clinic for a non‐traumatic fracture of the left femur 664 days after the RT initiation and was euthanised without further diagnostics (Grade V bone AE). No necropsy was performed. It was not possible to differentiate radiation‐induced bone necrosis vs. other aetiologies such as primary radiation‐induced or metastatic bone tumour as the cause of the fracture. Radiation dose to the femur (volume 79.1 cc) was D50% 55.6 Gy, D98% 51.1 Gy and D2% 57.5Gy. This event was recorded as a probable RT related AE [8].

Another dog with a recurrent, 20.9 cm longest diameter infiltrative lipoma extending from the left ventral abdomen to medial thigh received 2.6 Gy × 20 Fr (IMRT). A partial response to RT was documented along with transient acute skin AE (Grade III) and pain (Grade III), but diffuse oedema and serous discharge occurred within the RT field 121 days after the start of RT. No advanced imaging or cytological or histopathological assessment of the lesion was performed, and the dog was euthanised 227 days later due to progressive swelling without necropsy (Grade V skin AE). The radiation dose to the skin at the location of the AE could not be retrieved, but this event was clinically suspected as a probable RT AE [8].

The third case was treated with 3 Gy × 19 Fr (IMRT) for an incompletely excised infiltrative lipoma on the axilla. The dog developed progressive lameness in the ipsilateral forelimb. Radiographs were consistent with osteolysis of the proximal humerus and multifocal pulmonary nodules; the proximal humerus was within the irradiated field. The dog was euthanised shortly, 1697 days after the start of RT without further diagnostics (Grade V bone AE). Radiation dose to the humerus (volume of 79.6 cc) was D50% 51.9 Gy, D98% 1.6 Gy and D2% 59.8 Gy. While the lytic lesion was not histologically assessed, based on the clinical findings, this event was suspected to be a secondary malignancy and considered a definite RT‐related AE [8].

The fourth dog, a perianal infiltrative lipoma with pelvic and intra‐abdominal invasion, was treated with 2.5 Gy × 20 Fr (3DCRT) for gross disease, with stable disease as the best tumour response. Tumour progression was noted 1215 days after the completion of RT, and surgical resection was performed. The dog acutely died post‐operatively. Necropsy was consistent with focal acute jejunal perforation with fibrinosuppurative diffuse peritonitis. Radiation dose to the small intestine was D50% 12 Gy and D2% 42 Gy. Given the low dose distribution to the small intestine and the unlikely event that the same jejunal loop was included in each fraction, this event was considered unlikely related to RT [8].

The fifth dog was treated with 3 Gy × 19 Fr RT (IMRT) for an incompletely excised infiltrative lipoma in the right medial thigh. Tumour progression was not observed; however, the dog developed urothelial carcinoma of the urethra and was euthanised 1483 days after RT. Planned urethral dose was D50% 26.2 Gy and D2% 52.3 Gy. While the causality cannot be confirmed, this event was considered a Grade V AE (secondary malignancy) with attribution to RT deemed as possible [8].

The final dog was treated for gross disease on the right shoulder with 2.7 Gy × 20 Fr (IMRT). The dog had objective stable disease throughout the follow‐up period, but developed a solitary pulmonary carcinoma and underwent a lung lobectomy 1583 days after completing RT. The dog developed progressive respiratory distress postoperatively and was euthanised 2 days following surgery. Planned radiation dose to the lungs was D50% 15 Gy and D2% 52.7 Gy, with the region where the lung carcinoma developed receiving 15–30 Gy. This event was classified as a Grade V AE, possibly attributed to RT [8].

### 3.4 Outcome

Local disease progression was documented in four dogs (14%) based on physical examination ± CT 273, 423, 675 or 1215 days after RT. All four dogs received less than 51 Gy total dose including 2.7 Gy × 18 Fr, 2.5 Gy × 20 Fr, 2.5 Gy × 20 Fr and 4 Gy × 5 Fr protocols. Two underwent surgical resection approximately 21 or 43 months after RT due to disease progression and died post‐operatively due to complications, including a Grade V AE described above. No additional dogs received surgery or re‐irradiation of the tumour.

At the time of data analysis, 20 dogs were deceased and 8 dogs were alive with a median follow‐up duration of 1247 days (range = 198–2488 days). One dog was lost to follow‐up at the time of disease progression, 273 days after the start of RT. Among the 20 deceased dogs, the cause of death was attributed to the progression of infiltrative lipoma (*n* = 1) or radiation‐associated AE or secondary malignancies (*n* = 6) in seven cases. The dog that died due to progressive infiltrative lipoma developed sepsis/shock in the post‐operative period after resection of the recurrent infiltrative lipoma. The surgery was performed 21 months after RT (2.5 Gy × 20 Fr). Upon review of the case and because the underlying cause of sepsis was not determined, this was not considered an RT‐related AE. Causes of death in the other dogs included liver failure (*n* = 2), renal failure (*n* = 1), heart failure (*n* = 1), dog bite (*n* = 1), acute pancreatitis and pancytopenia (*n* = 1), pulmonary carcinoma (*n* = 1), bicavitary effusion with pulmonary nodules (*n* = 1), multicentric lymphoma (*n* = 1), radiation‐unrelated non‐traumatic femoral fracture (*n* = 1; RT given to infiltrative lipoma on an elbow), decreased mobility (*n* = 1), epistaxis of unknown cause (*n* = 1) and sepsis/shock (*n* = 1). The dog with sepsis/shock received RT for infiltrative lipoma on a limb, but the underlying cause of sepsis was not determined. The dog presented obtunded, laterally recumbent, and in shock, and was euthanised without further workup.

For the two dogs that received the 4 Gy × 5 Fr protocol for gross disease located on limbs extending to the trunk, the best reported tumour response was stable disease. One dog was lost to follow‐up at the time of local disease progression, 273 days after the start of RT. The other dog developed a pulmonary carcinoma and died post‐operatively following a lung lobectomy, 464 days after RT. Although the infiltrative lipoma remained stable in size throughout the follow‐up period, this dog initially presented with tenesmus, which resolved following RT.

### 3.5 Survival Analysis

**FIGURE 1:**
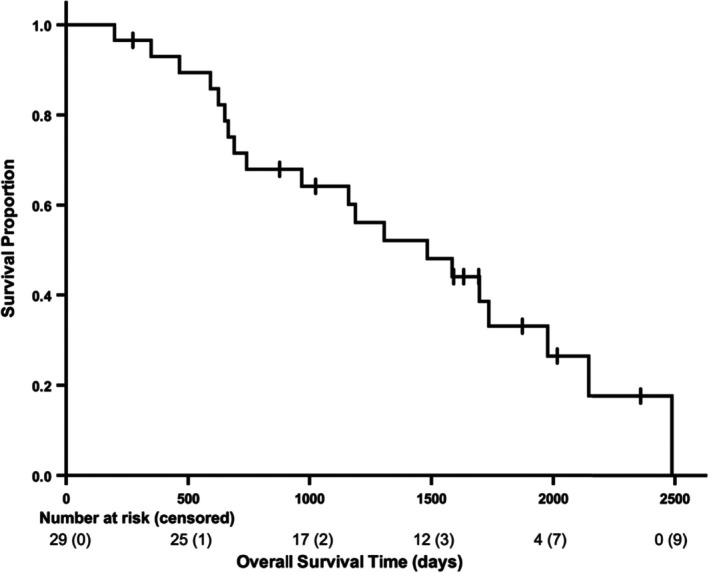
Kaplan–Meier curve for overall survival of all 29 dogs treated with radiation therapy for infiltrative lipoma. The median overall survival time was 1483 days (95% confidence interval = 853–2113 days; range = 198–2488 days). Hatch marks indicate censors (*n* = 9).

**FIGURE 2:**
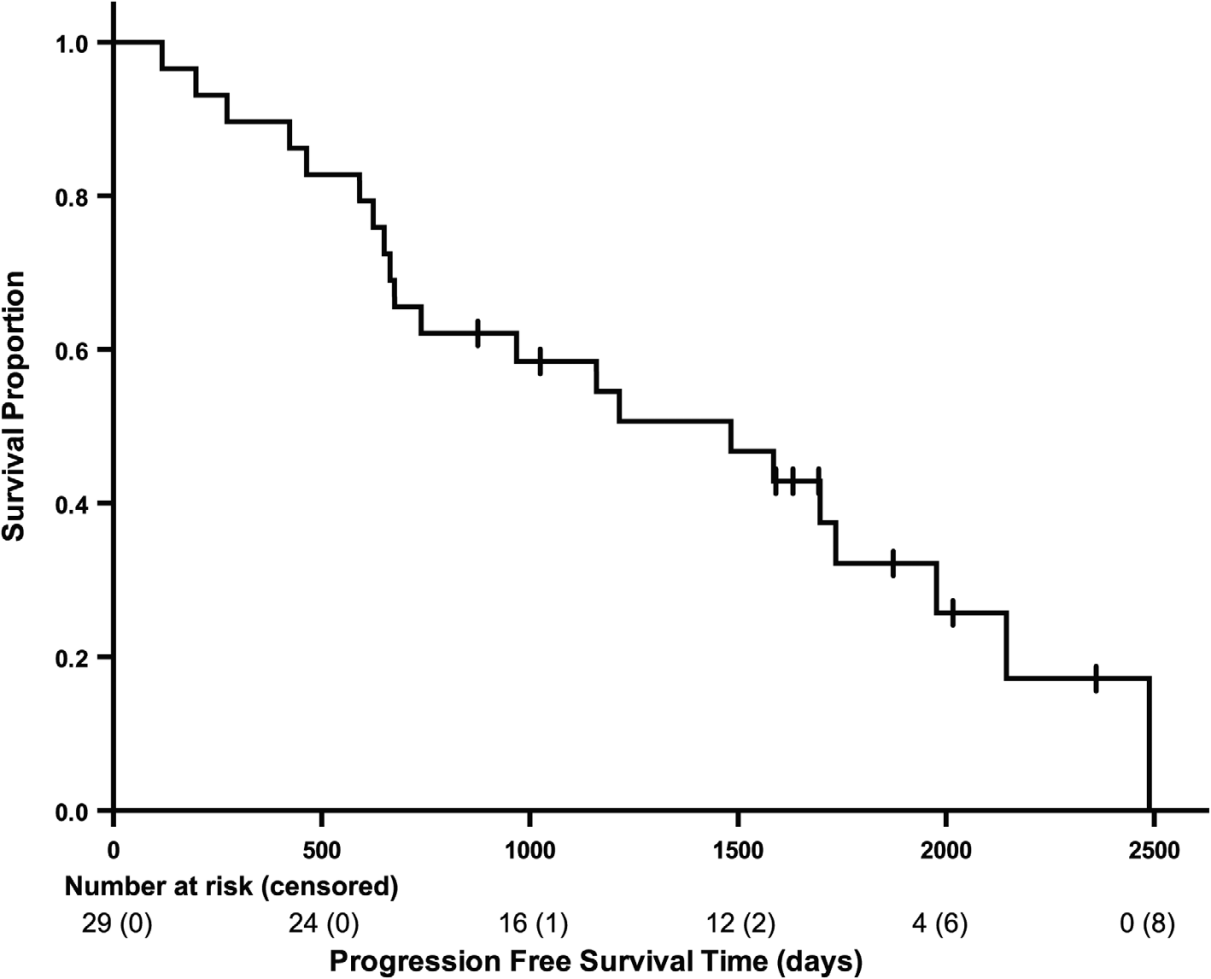
Kaplan–Meier curve for progression‐free survival of all 29 dogs treated with radiation therapy for infiltrative lipoma. The median progression‐free survival time was 1483 days (95% confidence interval = 745–2221 days; range = 117–2488 days). Hatch marks indicate censors (*n* = 8).

The median OS and PFS of the 29 dogs were 1483 days (95% confidence interval [CI] = 853–2113 days; range = 198–2488 days; Figure 1) and 1483 days (95% CI = 745–2221 days; range = 117–2488 days; Figure 2), respectively. The eight alive dogs and one dog that was lost to follow‐up were censored from OS analyses, while only the eight alive dogs were censored from the PFS analysis. Among the seven dogs that underwent RT as a sole therapy without prior surgery, disease progression was documented in two dogs, and neither dog underwent additional therapy. The median OS and PFS of these seven dogs were both 2488 days (95% CI = uncalculatable; range = 273–2488 days).

Log‐rank tests showed no significant difference in survival time between dogs with microscopic and macroscopic disease (OS *p* = 0.96; PFS *p* = 0.94), with and without symptoms at the time of RT (OS *p* = 0.4; PFS *p* = 0.67), comorbidities (OS *p* = 0.33; PFS *p* = 0.4), or tumour location (OS *p* = 0.16; PFS *p* = 0.06), or among dogs with a different number of previous surgeries (OS *p* = 0.07; PFS *p* = 0.13).

**FIGURE 3:**
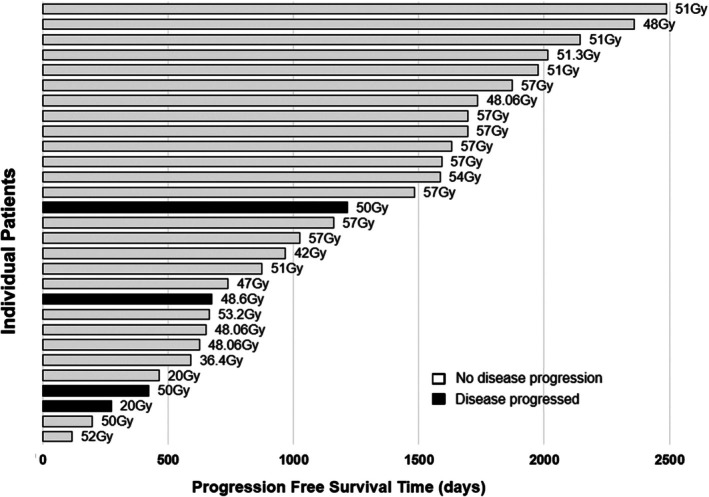
Progression‐free survival (days) for individual patients treated with various radiation doses. Each horizontal bar represents the progression‐free survival time of a single patient. The radiation dose delivered to each patient is indicated at the end of each bar in Gy. Dogs are grouped by their clinical outcomes, as indicated by the shading and pattern of the bars: grey bars, dogs with no evidence of disease progression during the study period (*n* = 25); and black bars, dogs with no evidence of disease progression during the study period.

Univariate Cox regression analysis showed that total radiation dose, GTV and number of previous surgeries were factors for OS with *p* < 0.1. In multivariate analysis, only total radiation dose remained significant (Figure 3). As the total dose increased by 1 Gy, the risk of death decreased by 10% (hazard ratio [HR] = 0.9; 95% CI = 0.839–0.966; *p* < 0.01). When GTV data were analysed only for the 23 dogs with macroscopic disease, GTV also remained significant in multivariate analysis (*p* < 0.01). For PFS, total radiation dose, GTV and tumour location were identified as risk factors in univariate analysis, but GTV was the only significant variable in multivariate analysis (*p* = 0.03; Table 5). The significance of GTV as a risk factor remained even when only the 23 dogs with macroscopic disease were analysed (*p* = 0.01).

**TABLE 5:** Factors evaluated for impact on overall and progression‐free survivals by Cox proportional hazards model multivariate analysis.

Factor	Overall survival		Progression‐free survival	
	*p*	HR (95% CI)	*p*	HR (95% CI)
Total radiation dose	< 0.01	0.9 (0.839–0.966)	0.22	0.953 (0.884–1.029)
GTV (*n* = 29)	0.14	1.001 (1–1.002)	0.03	1.001 (1–1.003)
GTV (*n* = 23)^a^	< 0.01	1.002 (1.001–1.004)	< 0.01	1.004 (1.001–1.006)
Number of surgeries	0.26	N/A	N/A	N/A
Tumour location	N/A	N/A	0.62	N/A

Abbreviations: 95% CI, 95% confidence interval; GTV, gross tumour volume; HR, hazard ratio; N/A, not applicable.

^a^ Includes only dogs with gross disease at time of RT.

When all 29 dogs were stratified based on the median GTV of 154.21 cm^3^, there was no difference in OS (*p* = 0.53) or PFS (*p* = 0.83). Similarly, when the 23 dogs with macroscopic disease at the time of RT were analysed separately and stratified using a GTV cut‐off of 145 cm^3^, there were no significant differences between the two groups in OS (*p* = 0.91) or PFS (*p* = 0.71). The cut‐off value was lower since GTV for microscopic disease (*n* = 6) was contoured on surgical incision with hemoclips (median = 201.4 cm^3^). When all 29 dogs were stratified based on the median prescribed total dose of 51 Gy, dogs that received 51 Gy or higher (*n* = 16) had significantly longer OS (median = 1977 days; 95% CI = 1476–2478 days vs. 739 days; 95% CI = 591–887 days) than dogs prescribed less than 51 Gy (*n* = 13) (*p* < 0.01). Likewise, PFS was significantly longer in dogs prescribed 51 Gy or higher compared to those prescribed less than 51 Gy (median = 1977 days; 95% CI = 1476–2478 days vs. 650 days; 95% CI = 551–749 days; *p* < 0.01). This difference remained significant when the two dogs that received 4 Gy × 5 Fr protocol were excluded (OS *p* < 0.01; PFS *p* < 0.01).

## 4 Discussion

This retrospective study evaluating outcomes in 29 dogs with infiltrative lipoma supports that excellent long‐term tumour control may be achieved with the incorporation of RT into a therapeutic approach. This result is consistent with previous literature and supports the use of RT in the clinical management of infiltrative lipoma in dogs [4, 5, 6]. The median OS in this population was greater than 4 years, with 4 dogs (14%) developing progressive disease (at a median or mean of 549 days). Seven dogs died secondary to disease (*n* = 1) or RT‐related AEs or secondary malignancy (*n* = 6) during the follow‐up period. In previous studies, the tumour‐associated death rate of canine infiltrative lipoma treated with RT is as low as 0%–14% (Table 6). This study required a 2‐year follow‐up time to account for the slow progression of canine infiltrative lipomas so that mature data was reported. This likely contributed to the capture of late radiation‐induced AEs and secondary malignancies, which occurred in greater numbers than deaths due to disease.

**TABLE 6:** Summary of previous and current studies of dogs with infiltrative lipoma treated with radiation therapy.

Study	Radiation protocol		*n*	Outcome		
	Dose (range, per fraction)	Median		Local control	Median OS	Tumour‐related death rate
McEntee et al. [4]	45.6–63 Gy; 2.5–4 Gy/Fr	54 Gy	13	92% (12/13)	1200 days	8% (1/13)
Feng et al. [5]	45–51 Gy; 2.4–3 Gy/Fr	48 Gy	24	92% (22/24)^a^	1760 days	0% (0/24)
Hauser et al. [6]	45–54 Gy; 3 Gy/Fr	48 Gy	21	76% (16/21)	1694 days	5% (1/21)
Current study	20–57 Gy; 2.4–4 Gy/Fr	51 Gy	29	86% (25/29)	1483 days	14% (4/29)

Abbreviations: Fr, fraction; OS, overall survival.

^a^ A new mass within the RT field but without pathology was considered a recurrence (*n* = 1).

For dogs with inoperable disease, RT was effective in the management of infiltrative lipoma as a first‐line therapy without surgery. Although measurable tumour responses were not reliably recorded due to inconsistencies in assessment methods, limited response documentation, and potential selection bias in follow‐up evaluations, the PFS of the seven dogs treated with RT as a sole modality was greater than 6 years. Interestingly, the number of prior surgeries and presence or absence of macroscopic disease did not affect the PFS or OS. These findings support the results of the recent study by Feng et al., where the number of surgeries and disease status were not prognostic for outcome [5]. This contrasts with the study by Hauser et al., in which an increased number of surgeries was associated with early disease progression [6]. Since our cohort included only two dogs that underwent more than two surgeries, it is possible that we did not find a difference due to a Type II error. Recurrent tumours may be more invasive and difficult to treat effectively with RT due to the larger treatment field or due to challenges related to the accurate identification of the extension of disease post‐operatively. Further investigation is needed to determine the role of multiple surgeries in tumour control after RT.

Our study is the first to demonstrate an association between higher (51 Gy or higher) prescribed total RT doses and improved PFS and OS. Caution is warranted as 51 Gy represented an arbitrary cut‐off and may not be representative of a threshold dose for improved control. It stands to reason that higher radiation doses may be associated with improved control, given that most of the dogs in this study had large, macroscopic tumours ranging from 5 to 2534 cm^3^ with a median GTV of 145 cm^3^. Further studies are needed to clarify the optimal dose threshold for improved tumour control and survival in canine infiltrative lipomas.

Our cohort included two dogs treated with the 4 Gy × 5 Fr protocol. The best reported tumour response in both cases was stable disease. These cases were included despite the palliative intent of the fractionation regimen, to evaluate a dose–response relationship within this cohort, and due to the lack of a standardised dosing regimen for this slowly progressive, benign histologic subtype. One dog experienced disease progression 273 days post‐RT, while the other dog died from surgical complications related to pulmonary carcinoma 464 days after RT, with no documented disease progression. In the latter dog, RT resulted in durable resolution of defecation problems related to intrapelvic extension of the infiltrative lipoma for which the dog originally presented. Although the small sample size precludes drawing definitive conclusions about the efficacy of this protocol, this palliative‐intent protocol may represent a reasonable option for further investigation as an alternative approach that is less time intense, less expensive and could theoretically be repeated in the event of progressive disease. Notably, neither of these dogs developed acute AE, and chronic AE was limited to Grade I leukotrichia in one case.

We anticipated that the incidence of acute AEs would be relatively high, given the high total RT doses prescribed to dogs in a definitive‐intent manner for long‐term tumour control. While most of the acute AEs were mild to moderate (Grade I or II), more severe AEs (Grade III or IV) occurred in over 20% of patients. These findings are consistent with Hauser et al., who also observed a high incidence of acute AEs [6]. While Feng et al. reported a relatively lower rate of acute AEs, this discrepancy may be partly due to differences in radiation dosing, protocol design, treatment planning (dose to OARs) or variations in the reporting and grading of AEs across studies [5]. Because none of the studies have been prospectively conducted, it is likely that all suffer from underreporting or underestimating the true AE incidence. This possibility highlights the need for clinicians to ensure comprehensive discussions regarding the potential risks and benefits of RT take place with pet owners. Discussions should include the likelihood for acute AEs, management strategies, possible protocol deviations that may be necessary during treatment, and the possibility of serious late AEs, which generally occur years following treatment.

In our study, six dogs (21%) died due to Grade V radiation AEs or secondary malignancies between 122 and 1697 days. Those dogs were all prescribed total doses of 50–57 Gy. Two of these dogs were euthanised for appendicular bone changes in the irradiated field. Planned dose to irradiated bones included D50% 55.6 and 51.9 Gy, and D2% 57.5 and 59.8 Gy, respectively. Because further diagnostics or post‐mortem were not available, it was unclear if this represented radiation‐induced osteoradionecrosis or radiation‐induced secondary tumour formation. Either way, this highlights the potential risk of late, severe AEs associated with higher total doses to OARs when survival time after RT is long. Owners should be informed about the possibility of secondary tumour formation. Six out of 29 dogs (21%) in this cohort that experienced such events represents a meaningful and unexpected proportion of life‐threatening complications. Bones and other normal surrounding structures should be contoured and optimised as OARs to minimise late AEs, especially given that dogs undergoing RT for infiltrative lipoma can survive for several years. Indeed, in human patients receiving fractionated RT, applying dose constraints has been shown to reduce the 5‐year risk of RT‐induced bone fractures [12]. Larger treatment fields and the use of 3D conformal radiation planning as opposed to intensity modulated techniques may increase the likelihood of including critical structures within the high‐dose region, thus elevating the risk of both acute and chronic AEs.

In this study, malignancies that developed within the RT field were carefully reviewed by the authors and included as AEs. None would have been considered Grade V AEs per VRTOG v1 [13]. In line with recommendations in VRTOG v2, we classified all deaths as Grade V AEs when planned dose existed within the region of the AE [8]. The attribution was provided to clarify the likely contribution from RT. The relatively high prevalence of Grade V AEs in our population is likely caused by this inclusion and the long‐term follow‐up achieved in our cases.

Due to its retrospective design, several limitations require attention, including the lack of standardised radiation planning and delivery techniques, prescription and monitoring protocols, and tumour response evaluation. Another key limitation is the absence of a control group, making it difficult to definitively assess the effects of RT alone. It is worth noting that 80% (23/29) of the dogs in our cohort were treated with RT for gross disease, and 55% (16/29) received RT following surgery for recurrent lipomas. While canine infiltrative lipomas are known to progress slowly, these findings suggest that RT played an important role in controlling disease progression and improving outcomes, particularly in cases where surgical excision was incomplete or recurrence occurred. Retroactive application of the VRTOG toxicity criteria may have resulted in inaccurate reporting of radiation‐related AE. Finally, given the small sample size, the lack of statistical significance in the multivariate analysis can be a Type II error. Given these limitations, the interpretation of our results should be approached with caution. Prospective studies can build on data presented here to study the influence of tumour volume and radiation dose and technique so that guidelines may be established for the management of canine infiltrative lipomas.

In conclusion, the findings of this study support the use of RT as an effective treatment option for unresectable or incompletely excised canine infiltrative lipomas. The results suggest that a higher total radiation dose may improve long‐term local tumour control. However, the optimal dosing remains to be fully determined. Dose to OARs should be limited as much as possible considering the longer survival times achieved after RT. Further investigation of the palliative‐intent 4 Gy × 5 Fr protocol in a larger cohort of dogs is needed to define the therapeutic benefit given its advantages with respect to time, cost and AE risk. Given the limitations of this retrospective study, a prospective study with standardised treatment protocols and long‐term follow‐up is recommended to validate our findings and to further clarify the role of RT in managing this condition. Such studies will help optimise RT protocols, balancing efficacy with the risk of AEs, and ultimately improve patient outcomes.

## Conflicts of Interest

The authors declare no conflicts of interest.

## Supporting information


**Table S1.** Organ at risk doses.

## Data Availability

The data that support the findings of this study are available from the corresponding author upon reasonable request.
