# Supplementary material for: Outcome and Prognostic Factors of Dogs Treated for Infiltrative Lipoma Undergoing Radiation Therapy: A Retrospective Multi‐Institutional Study of 29 Cases
Source: Vet Comp Oncol. 2025 May 14;23(3):401–11. doi: 10.1111/vco.13065 (PMC12378080; doi:10.1111/vco.13065)
Supplement: Supplementary file 1 — Table S1. Organ at risk doses. [file VCO-23-401-s001.docx]

**Supplementary Table 1**. Organ at risk doses

|  | **Median (SD)** | | |
| --- | --- | --- | --- |
| **Organ (n)** | **D50%, Gy** | **D2%, Gy** | **Vprescription dose, %** |
| Spinal Cord (n=19) | 4.2 (12.1) | 26.5 (14) | 0 (0) |
| Lung (n=13) | 6.9 (6.3) | 49.8 (10.9) | 0 (1.1) |
| Heart (n=11) | 5.9 (8.3 | 25 (17.6) | 0 (0) |
| Rectum (n=8) | 17.4 (17.5) | 33.0 (17.6) | 0.5 (10.3) |
| Urethra (n=8) | 22.7 (11.6) | 45.3 (14) | 0 (4.2) |
| Bladder (n=7) | 14.1 (17.7) | 44.4 (15.1) | 0.5 (6.7) |
| Colon (n=7) | 12.1 (4.6) | 30.8 (11.7) | 0 (2.4) |
| Brain (n=4) | 12.0 (12.2) | 27.5 (12.3) | 0 (0) |
| Esophagus (n=4) | 11.5 (9.5) | 35 (17.3) | 0 (0) |
| Liver (n=4) | 16.75 (9.3) | 50.9 (12) | 1.4 (1.6) |
| Trachea (n=4) | 4.71 (17.4) | 22.0 (21.3) | 0 (1) |
| Right eye (n=3) | 4.3 (4.2) | 8.9 (12.2) | 0 (0) |
| Left eye (n=3) | 10.7 (6.8) | 20 (15.8) | 0 (0) |
| Bone (n=1) | 13.5 | 42.8 | 7 |
| Kidney (n=1) | 9.8 | 13.4 | 0 |
| Small intestines (n=1) | 12 | 42 | 0 |
| Spleen (n=1) | 19.2 | 46.7 | 0 |
| Vulva (n=1) | 41.1 | 50.6 | 4 |

Data on dosing to the skin was not collected.
